# A pilot dose–response study of the acute effects of haskap berry extract (*Lonicera caerulea* L.) on cognition, mood, and blood pressure in older adults

**DOI:** 10.1007/s00394-018-1877-9

**Published:** 2018-12-10

**Authors:** Lynne Bell, Claire M. Williams

**Affiliations:** School of Psychology and Clinical Language Sciences, University of Reading, Earley Gate, Whiteknights, Reading, RG6 6AL UK

**Keywords:** Haskap berry, Nutrition intervention, Older adults, Cognition, Blood pressure

## Abstract

**Purpose:**

Haskap (*Lonicera caerulea* L. or blue honeysuckle) is a plant native to the low-lying wet areas and mountains of Siberia and northeastern Asia, but is now cultivated in Canada. The dark blue berries are rich in anthocyanins, particularly cyanidin-3-*O*-glucoside. Previously, anthocyanin-rich fruits have been observed to benefit cognitive performance during the immediate postprandial period following a single acute dose. However, no study has currently examined the potential for haskap berries to influence cognitive performance. Here, we investigate the acute cognitive benefits of an anthocyanin-rich haskap berry extract.

**Methods:**

A double-blind, counterbalanced, crossover intervention study compared the acute effects of three separate haskap berry extract doses, containing 100 mg, 200 mg, and 400 mg anthocyanins, with a sugar-matched placebo. Participants were an opportunity sample of 20 older adults, aged 62–81 years. Measures of cognition, mood, and blood pressure were recorded at baseline and 1.5 h postprandially.

**Results:**

Compared to placebo, the 400 mg dose elicited significantly lower diastolic blood pressure and heart rate. Both 200 mg and 400 mg doses elicited significantly higher word recall, with the 400 mg dose also significantly improving word recognition scores, on an episodic memory task. However, mood, working memory and executive function task results were more equivocal.

**Conclusions:**

The findings provide evidence for improvements in episodic memory and blood pressure following acute supplementation with haskap berry extract, with higher doses appearing most effective. The cognitive findings concur with previous literature that suggests episodic memory effects, and not executive function effects, are most prevalent in older adults following anthocyanin-rich berry supplementation. The blood pressure outcome is consistent with a vasodilatory mechanism of action.

## Introduction

A rise in the incidence of age-related neurodegenerative diseases, and associated health care costs, is predicted to result from the ageing demographic of the UK population. Much current government thinking is, therefore, focused on the promotion of lifelong health, with a view to mitigating age-related cognitive changes in healthy older adults. Moreover, recently published National Institute for Health and Care Excellence (NICE) guidelines [1] recommends the development and support for population-level initiatives to reduce the risk of dementia, disability, and frailty by making it easier for people to stop smoking, be more physically active, reduce their alcohol consumption, maintain a healthy weight and, importantly for this research, to adopt a healthy diet. However, the efficacy of such dietary interventions on the cognitive ageing process is currently under-investigated.

Flavonoids are a class of organic polyphenol micronutrients that are found in high concentrations in plant-based foods such as berries, tea, cocoa, and whole grains. Flavonoids have been linked to improvements in cardiovascular and metabolic health and are also showing particular promise in terms of their effect on memory and other aspects of cognition. In recent years, a large body of evidence has emerged from human intervention studies demonstrating that the consumption of flavonoid-rich foods is associated with improvements in cognitive function [2–5]. Specifically, flavonoids (cocoa flavanols, citrus flavanones and berry anthocyanins) have been observed to exert positive effects on executive functioning and memory in both older adults [6, 7] and children [8–10]. Thus, dietary interventions rich in flavonoids may represent a plausible, and publically acceptable, route for the amelioration of age-related cognitive decline. Indeed in a recent study of older adults, blueberry extract was observed to significantly improve episodic memory and visuospatial working memory after 3-month supplementation, and to reduce systolic blood pressure after both 3- and 6-month supplementation, when compared to a control group of participants [11].

Much of the flavonoid-based cognitive research to date has focussed on blueberries, citrus fruits, cocoa, and green tea; however, other berries including blackcurrants, grapes and cherries are increasingly under investigation due to their high flavonoid content [2]. Haskap (*Lonicera caerulea* L. or blue honeysuckle) is a plant native to the low-lying wet areas and mountains of Siberia and northeastern Asia. The fruit from this plant, haskap berries or honeyberries, are elongated berries covered in a dark blue to purple skin. Importantly, it has recently been ascertained that the haskap berry is particularly rich in the flavonoid anthocyanins and may, therefore, represent an, as yet, unexplored dietary intervention for neurocognitive health in older adults. The average anthocyanin content of haskap berries is 1080 mg per 100 g fresh weight of the fruit [12] which compares very favourably with other fruit sources such as raspberry (50 mg/100 g), blackberry (100 mg/100 g), red currant (75 mg/100 g) and blueberry (150 mg/100 g) [13]. A number of in vitro and in vivo studies have assessed the potential health benefits associated with consumption of haskap berries with data showing potential application as anti-microbial, anti-cancer and anti-inflammatory agents [14]. However, despite the high anthocyanin content of the fruit, no studies have assessed the impact of haskap supplementation on cognitive performance. This pilot study, therefore, examined the effectiveness of a haskap berry intervention for improving cognitive performance in older adults. In an acute study we compared the effects of several different doses of freeze-dried haskap berry extract on cognitive performance in older adults, with the aim of identifying the optimal dose for acute neurocognitive change in this age group.

## Materials and methods

### Participants

Twenty older adults, aged 62–81 years (mean 70.50, SD 5.49, 9 male), were recruited from the local area via opportunity sampling. Ethnicity was not formally recorded, but participants were predominantly of white British descent. Sample size was determined by power analysis using GPower 3.1. Assuming a medium effect size [2], 20 participants were needed to detect a significant difference between repeated dose conditions with an alpha of 0.05 and 80% statistical power. BMI ranged from 16 to 36 kg/m^2^ (mean 24.94, SD 4.54). Habitual consumption of fruits and vegetables ranged from 3 to 9 daily portions (mean 5.58, SD 1.76). Participants reported being generally healthy, were non-smokers, and were free from diabetes. Regular doses of medications commonly used in this age group, such as statins or anti-hypertensives, were permitted during the trial; however, those taking anti-depressant or anti-coagulant medications, or irregular doses of other medications were excluded.

### Cognitive measures

The selected tasks have all been used in previous crossover nutrition intervention studies [2] and included measures of working memory (serial 3s and 7s subtraction), attention/executive function (Attention Network Task, ANT), and episodic memory (Auditory Verbal Learning Task, AVLT). The tasks were all programmed using E-Prime. To minimise the impact of cognitive practice effects on the study outcome [15], the first visit was treated as a familiarisation visit where participants received instruction on the tasks and became familiar with the study procedure. During this visit, participants completed two practice sessions of the full task battery but no data were collected. At subsequent visits, short practice trials were incorporated at the beginning of each executive function or working memory task, for which data were again not collected. Alternate forms of stimuli were used for repeat presentations of all tasks, with randomisation across visits.

#### AVLT

This episodic memory task was a multi-trial word list learning task with immediate and delayed free recall, and delayed recognition components [16]. Participants listened to a recording of 15 words and were immediately asked to verbally recall as many as possible within a 2-min time period. The same word list (List A) was repeated a further four times, each followed by a recall period (R1–R5). Another unrelated list of 15 words was then introduced (List B). Participants verbally recalled List B (RB) before again recalling List A (R6). After a delay of approximately 15 min, during which time the remaining cognitive tasks were completed, participants again verbally recalled List A (R7), then performed a visual recognition task selecting List A words from a list of 50 words also containing 15 List B foils and 20 previously unseen words. Different word lists, matched for concreteness and familiarity, were used at each test session [10]. The dependent variables were the total number of correct words recalled/recognised at each repetition, plus calculated measures of proactive (R1–RB) and retroactive interference (R5–R6), and learning (R5–R1) [16].

#### Serial subtraction, 3s and 7s

This working memory task used a previously published method [17–19], whereby a random number between 800 and 999 was presented on screen and participants counted backwards, entering their answers using the computer number pad. Participants were required to enter their responses as quickly as possible for a total of 2 min. The task was first performed subtracting 3s and was then repeated subtracting 7s. The dependent variables for both 3s and 7s were the total number of correct responses and the total number of errors recorded in 2 min. The accuracy of each response was determined relative to the previous response, whether or not the previous response was correct.

#### ANT

This executive function task combines the Eriksen flanker task [20] with the Posner spatial cueing task [21] to investigate multiple facets of attention: alerting, orienting, and executive attention [22]. Participants viewed blocks of arrows presented on screen in rapid succession and indicated the direction of the arrow closest to a central fixation point with a key press (left or right arrow). The target arrow was either flanked by arrows pointing in the same (congruent) or opposite (incongruent) direction, or was not flanked at all. On selected trials, spatial cues were introduced immediately prior to the appearance of the arrows. Task load was further manipulated by increasing or decreasing the number of flanking arrows across multiple trials [9]. The dependent variables were reaction time and accuracy by congruency, cue, and load.

### Mood measures

The PANAS-Now [23] mood questionnaire, where participants rate 20 mood adjectives on a scale of 1–5, and a subjective nine-point Likert scale measure of mental fatigue [17] were administered at the end of each session of cognitive tasks. Dependent variables were mental fatigue score, and composite positive affect and negative affect scores obtained by summing responses to positive or negative PANAS items, respectively. PANAS items relating to attention (‘alert’ and ‘attentive’) were also analysed individually, as anthocyanin-rich berry interventions have previously shown positive effects on attention [24, 25].

### Blood pressure and heart rate

Blood pressure and heart rate measurements were recorded using a clinically validated Omron M6 Comfort automatic digital upper arm blood pressure monitor. Three repeat readings were taken at 2-min intervals and average values recorded, in accordance with the manufacturer’s instructions. To maintain consistency between repeat test sessions, blood pressure measurements were recorded in a seated position, with the cuff placed on the left arm, and with the arm resting on an adjacent desk throughout the measurement. Blood pressure was recorded at the end of the test battery when participants had been seated for 25 min.

### Other measures

#### Palatabilty

Palatability of each dose was determined immediately after consumption by a nine-item Likert scale questionnaire where participants rated various taste dimensions (sweet, sour, bitter, pleasant) on a scale of 1–9.

#### Fruit and vegetable consumption

At the practice visit, participants were given portion size definitions for a range of different fruit and vegetable types and were asked to estimate their average daily consumption of fruits and vegetables.

#### Body mass index

Weight and height measurements were recorded for each participant at the beginning of the practice visit. These were used to calculate BMI using the formula: BMI = weight (kg)/height (m)^2^.

### Haskap berry interventions

The intervention was a commercially available haskap berry extract, supplied by Global Direct Commodities Limited, Canada. Obtained by alcohol extraction and then freeze-dried, the extract was standardized to contain 25% anthocyanins. Anthocyanin profile and total polyphenol content were not quantified. Varying doses of haskap berry extract powder were mixed with low-flavonoid lemon squash (Rocks Organic), sugars, and water. Exact quantities are shown in Table 1.

**Table 1:** Intervention drink composition

	Control	Low	Medium	High
Extract (mg) (total anthocyanins^a^)	0 (0)	400 (100)	800 (200)	1600 (400)
Fructose (g)	10	10	10	10
Glucose (g)	10	10	10	10
Lemon squash (ml)	30	30	30	30
Water (ml)	220	220	220	220

^a^Extract standardized to contain 25% anthocyanins

### Procedure

**Fig. 1:**
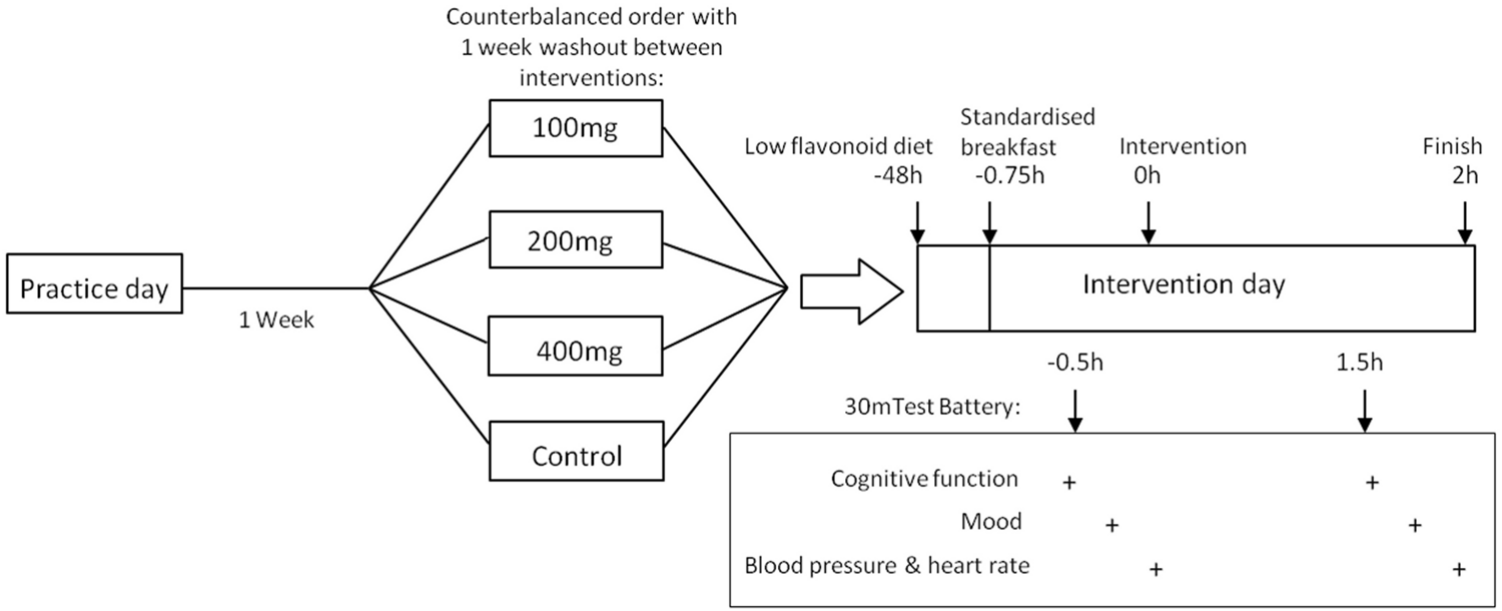
Study design

The double-blind, crossover study design is illustrated in Fig. 1. Participants attended the lab on five separate occasions each separated by 1 week with a different dose of intervention administered, in counterbalanced order, on each occasion. For 24 h prior to each visit, participants followed a low-polyphenol diet and were asked to keep a food diary of all food and drink consumed. They attended each visit 12 h fasted. The usual start time was 9 a.m., though some participants opted to attend up to 1 h earlier or later. On arrival they received a standardized low-polyphenol breakfast of croissants and cream cheese, with water to drink. Fifteen minutes was allowed for breakfast. Participants then completed a 30-min test battery consisting of cognition, mood, and blood pressure measures. Testing took place in the following order: AVLT (immediate recall), serial 3 s and serial 7 s subtraction, ANT, AVLT (delayed recall and recognition), mental fatigue and PANAS, blood pressure and heart rate.

The intervention was administered in the form of a 250 ml beverage, served chilled (5 °C) in an opaque cup, and consumed through a black straw to maintain double-blinding. Participants were asked to consume the drink as quickly as possible within a maximum 15-min period. After consuming the drink, participants completed a palatability questionnaire and waited for a total of 90 min before repeating the test battery. Peak plasma concentrations of anthocyanin metabolites accompanied by a peak in vascular response have previously been observed between 1 and 2 h postprandially [26]; therefore, the test battery was timed to fall within this period. During the break, participants were supplied with magazines to read. After testing had been completed, a return appointment was made for the following week. At the end of the study participants received a £25 payment and a jar of haskap berry jam.

### Data analysis

All data were analysed using IBM SPSS statistics version 22. Parametric tests were used throughout after confirming that test data met the required assumptions of normality. For all RT data, only correct responses were included in mean values. *Z* score analysis was used to identify outliers; data points with *z* score > 3.29 were removed prior to statistical analysis [27]. A linear mixed model (LMM) using an unstructured covariance matrix to model successive repeat test sessions was used to analyse data for all measures. Baseline performance was included as a repeated covariate, and age, BMI, and habitual fruit and vegetable consumption were included as demographic covariates, due to their variability amongst this opportunity sample and the known impact on physiology and cognitive function. Visit and dose were included as fixed factors in the model (visit was included to model any residual practice effects [15]). The aim of the analysis plan was to determine whether haskap berry dose was a significant predictor of cognition/mood performance, or blood pressure. Post hoc pairwise comparisons were used to investigate any significant effects of dose. A Bonferroni correction was applied to all multiple comparisons. Significant comparisons are reported (*p* < 0.05). For the ANT task only, the additional factors of congruency, cue type, and load, and their respective dose interactions, were added to the LMM analysis.

## Results

Following publication, data supporting the results reported in this paper will be openly available from the University of Reading Research Data Archive at http://researchdata.reading.ac.uk/.

Data were successfully collected for 19 participants. Due to an administrative error, data for the post-intervention time point following the 200 mg dose were missing for one further participant; however, all other data for this participant have been included in the analysis.

### Visit order effects

Significant visit order effects were observed for mental fatigue [*F*(3,16.10) = 3.74, *p* = 0.033], serial 3s score [*F*(3,17.95) = 3.32, *p* = 0.043], serial 3s errors [*F*(3,17.53) = 3.45, *p* = 0.039], ANT accuracy [*F*(3,392.96) = 11.35, *p* < 0.001], and ANT RT [*F*(3,368.22) = 14.01, *p* < 0.001]. In general, cognitive performance was observed to improve over time, with higher scores, fewer errors and faster reaction times evident at later visits compared with earlier visits. In contrast, ratings of mental fatigue worsened over time.

### Blood pressure and heart rate

No dose effects were observed for systolic blood pressure [*F*(3,41.39) = 1.54, *p* = 0.219]. However, significant dose effects were observed for diastolic blood pressure [*F*(3,34.79) = 3.85, *p* = 0.018] and heart rate [*F*(3,35.03) = 4.48, *p* = 0.009]. Pairwise comparisons revealed that both diastolic blood pressure and heart rate were significantly lower following the 400 mg dose when compared to the control (*p* = 0.012 and *p* = 0.020, respectively). Results are presented in Table 2.

**Table 2:** Mean (SE) blood pressure and heart rate following varying doses of haskap berry extract

Measure	Anthocyanin dose (mg)			
	0 (control)	100	200	400
SBP (mmHg)	125.53 (1.65)	123.35 (1.64)	122.71 (1.66)	123.07 (1.64)
DBP (mmHg)	75.50 (1.28)	74.32 (1.27)	73.73 (1.29)	72.84 (1.27)*
HR (bpm)	63.02 (1.07)	62.71 (1.06)	61.50 (1.07)	60.63 (1.06)*

Reported values are estimated marginal means with baseline, age, BMI, and habitual fruit and vegetable consumption as covariates

Statistical differences from control are indicated, *p < 0.05

#### AVLT

**Fig. 2:**
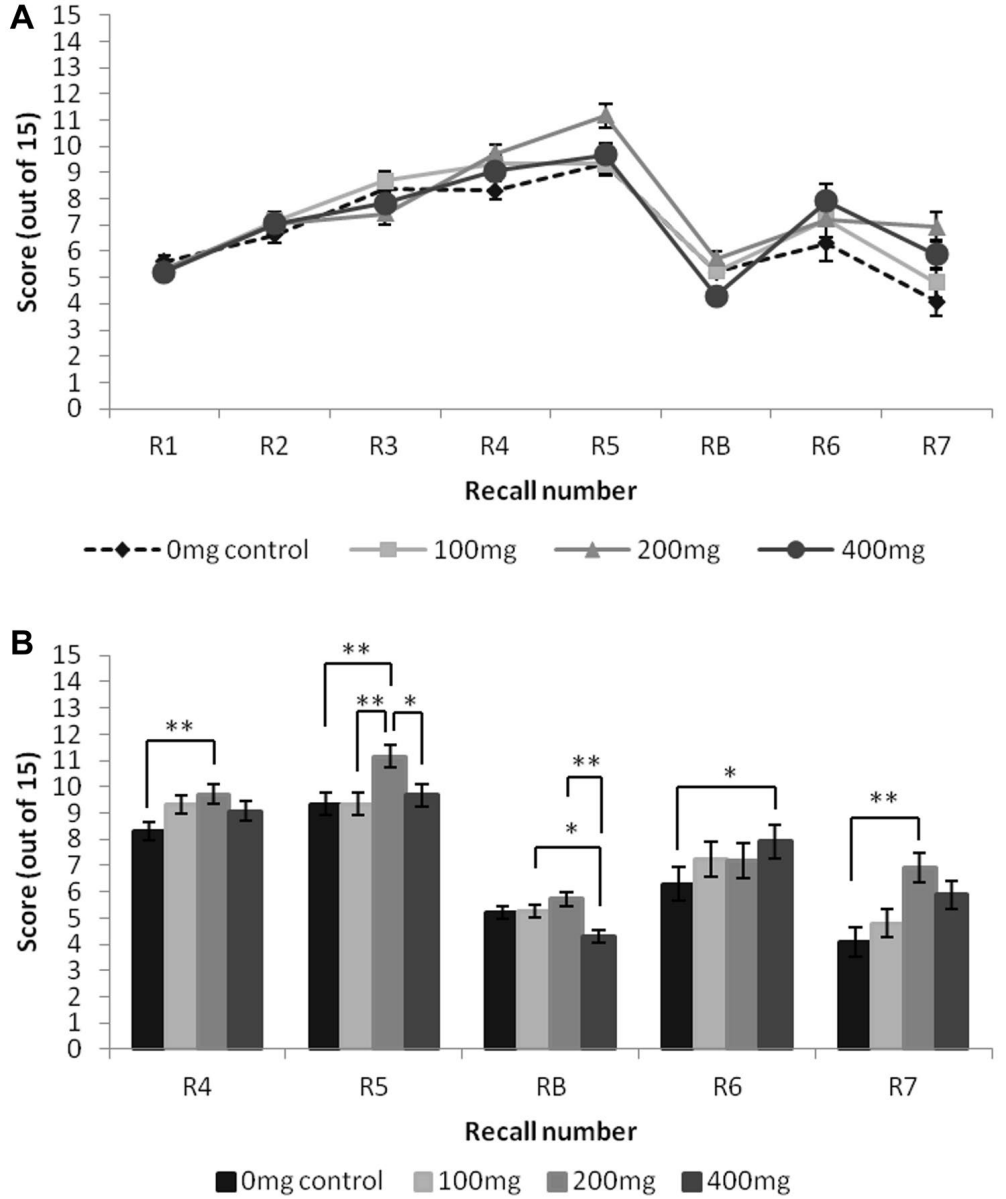
The effect of dose on AVLT word recall across multiple recall points. **a** Mean recall scores for all recall points following varying doses of haskap berry extract. **b** Recall points where statistical differences between dose conditions were observed. Reported values are estimated marginal means with baseline, age, BMI, and habitual fruit and vegetable consumption as covariates. Error bars represent standard error of the mean. Statistical differences are indicated, **p < 0.01, *p < 0.05

There were significant dose effects at recall points R4 [*F*(3,30.61) = 5.49, *p* = 0.004], R5 [*F*(3,34.99) = 7.34, *p* = 0.001], RB [*F*(3,32.29) = 6.82, *p* = 0.001], R6 [*F*(3,27.80) = 3.24, *p* = 0.037], and R7 [*F*(3,34.71) = 5.49, *p* = 0.003]. Pairwise comparisons revealed that no significant differences in recall performance were observed following the 100 mg dose, compared to the control. Performance following the 200 mg dose was significantly higher than the control at R4 (*p* = 0.004), R5 (*p* = 0.002), and R7 (*p* = 0.002). At R5, 200 mg dose performance was also significantly higher than the 100 mg and 400 mg doses, (*p* = 0.002 and p = 0.022, respectively). Performance following the 400 mg dose was significantly higher than control at R6 (*p* = 0.028). At RB, 400 mg dose performance was also significantly lower than the 100 mg and 200 mg doses, (*p* = 0.034 and *p* < 0.001), respectively. Results are presented in Fig. 2.

There were significant effects of dose for learning (R5–R1) [*F*(3,34.20) = 3.78, *p* = 0.019] and proactive interference (PI) (R1–RB) [*F*(3,16.58) = 6.01, *p* = 0.006], but not for retroactive interference (RI) (R5–R6) [*F*(3,31.96) = 0.78, *p* = 0.512]. Pairwise comparisons revealed no significant differences in proactive interference for any of the doses compared to control, although PI was observed to be lower following the 200 mg doses compared to the 400 mg dose (*p* = 0.033). Despite the significance of the learning main effect, there were no significant pairwise differences in learning between any of the doses.

For delayed recognition there was a significant effect of dose on the number of words correctly recognised [*F*(3,33.27) = 5.95, *p* = 0.002]. Pairwise comparisons revealed a significantly greater number of words recalled following the 400 mg dose compared to the control (*p* = 0.013). Results for AVLT calculated and recognition variables are presented in Table 3.

**Table 3:** Mean (SE) cognition scores following varying doses of haskap berry extract

Measure	Anthocyanin dose (mg)			
	0 (control)	100	200	400
AVLT (words)				
Learning	4.13 (0.42)	4.05 (0.43)	5.08 (0.43)	4.84 (0.43)
PI	0.36 (0.29)	− 0.22 (0.29)	− 0.37 (0.29)	0.82 (0.29)
RI	2.92 (0.36)	2.45 (0.36)	2.36 (0.36)	2.39 (0.36)
Recognition	10.09 (0.43)	10.48 (0.43)	11.45 (0.44)	11.51 (0.43)*
Serial subtraction (in 2 min)				
3s correct	34.18 (1.01)	32.01 (1.01)	35.15 (1.02)	31.89 (0.99)
3s errors	4.00 (0.40)	3.24 (0.39)	2.28 (0.41)**	3.07 (0.39)
7s correct	20.27 (0.82)	20.75 (0.84)	20.30 (0.85)	20.63 (0.82)
7s errors	3.42 (0.32)	2.00 (0.32)*	2.63 (0.31)	4.78 (0.32)*
ANT				
Accuracy (%)	0.97 (< 0.01)	0.97 (< 0.01)	0.97 (< 0.01)	0.98 (< 0.01)
RT (ms)	573.37 (2.22)	567.13 (2.21)	567.80 (2.27)	569.48 (2.22)

Reported values are estimated marginal means with baseline, age, BMI, and habitual fruit and vegetable consumption as covariates

Statistical differences from control are indicated, **p < 0.01, *p < 0.05

#### Serial subtractions

For serial 3s, significant dose effects were evident for number of correct responses [*F*(3,32.5) = 5.89, *p* = 0.002] and number of errors [*F*(3,31.41) = 5.21, *p* = 0.005]. Pairwise comparisons revealed no significant differences in number of correct responses compared to the control; however, performance following the 200 mg dose was significantly higher than both the 100 mg and 400 mg doses (*p* = 0.019 and *p* = 0.040, respectively). The number of errors was significantly lower following the 200 mg dose compared to the control (*p* = 0.002). For serial 7s, significant dose effects were evident for number of errors [*F*(3,6.11) = 27.73, *p* = 0.001], but not for number of correct responses [*F*(3,31.01) = 0.10, *p* = 0.957]. Pairwise comparisons revealed significantly fewer errors (*p* = 0.014) following the 100 mg dose, compared to the control. However, following the 400 mg dose, significantly more errors were made compared to the control (*p* = 0.011). Results are presented in Table 3.

#### ANT

For ANT accuracy, there was no significant effect of dose [*F*(3,871.61) = 0.99, *p* = 0.399] and no significant dose interaction with congruency [*F*(3,854.65) = 0.46, *p* = 0.711], cue type [*F*(9,854.50) = 0.476, *p* = 0.892], or load [*F*(3,855.22) = 1.15, *p* = 0.329]. Similarly for ANT reaction time, there was no significant effect of dose [*F*(3,777.25) = 1.97, *p* = 0.117] and no significant dose interaction with congruency [*F*(3,761.25) = 0.34, *p* = 0.794], cue type [*F*(9,759.52) = 0.73, *p* = 0.684], or load [*F*(3,759.47) = 1.55, *p* = 0.200]. Results are presented in Table 3.

#### PANAS and mental fatigue

There were no significant effects of dose on mental fatigue [*F*(3,34.46) = 0.66, *p* = 0.585], positive affect [*F*(3,32.95) = 0.53, *p* = 0.664] or negative affect [*F*(3,37.92) = 1.69, *p* = 0.185]. However, analysis of individual PANAS items relating to attention revealed a significant effect of dose on ratings of alertness [*F*(3,36.53) = 10.57, *p* < 0.001], but not attentiveness [*F*(3,36.97) = 0.90, *p* = 0.448]. Pairwise comparisons revealed significantly lower ratings of alertness following the 400 mg dose compared with the control (*p* = 0.028) and 100 mg dose (*p* < 0.001). Alertness following the 200 mg dose was also rated lower than the 100 mg dose (*p* = 0.0010). Results for all mood variables are presented in Table 4.

**Table 4:** Mean (SE) subjective mood ratings following varying doses of haskap berry extract

Measure	Anthocyanin dose (mg)			
	0 (control)	100	200	400
Mental fatigue (/9)	4.74 (0.35)	4.18 (0.35)	4.28 (0.36)	4.36 (0.35)
Positive affect (/50)	29.33 (0.80)	29.68 (0.81)	29.70 (0.81)	28.72 (0.80)
Negative affect (/50)	12.83 (0.50)	11.84 (0.51)	12.63 (0.52)	12.29 (0.51)
Alert (/9)	3.21 (0.17)	3.49 (0.17)	2.95 (0.17)	2.83 (0.17)*
Attentive (/9)	3.63 (0.13)	3.63 (0.13)	3.76 (0.13)	3.54 (0.13)

Reported values are estimated marginal means with baseline, age, BMI, and habitual fruit and vegetable consumption as covariates

Statistical differences from control are indicated, **p < 0.01, *p < 0.05

#### Palatability

There were significant effects of dose on subjective ratings of sweetness [*F*(3,38.85) = 8.86, *p* < 0.001], sourness [*F*(3,31.48) = 10.18, *p* < 0.001], bitterness [*F*(3,28.73) = 13.16, *p* < 0.001], and overall pleasantness [*F*(3,41.58) = 6.53, *p* = 0.001]. Pairwise comparisons revealed that compared to control, the 200 mg and 400 mg doses were rated significantly more sour (*p* = 0.002 and *p* < 0.001, respectively), and the 400 mg dose was also rated more sour than the 100 mg dose (*p* = 0.035). The 400 mg was rated significantly more bitter than the control (*p* = 0.001), the 100 mg dose (*p* < 0.001), and the 200 mg dose (*p* = 0.048). In terms of overall taste, only the 400 mg dose was rated significantly less pleasant than the control (*p* = 0.002) and was also rated less pleasant than the 100 mg dose (*p* = 0.035). For sweetness, there were no significant differences compared to control; however, the 200 mg and 400 mg doses were rated significantly less sweet than the 100 mg dose (*p* = 0.001 and *p* = 0.008, respectively).

## Discussion

The results demonstrate significant benefits of haskap berry extract on both physiological and cognitive outcomes in the immediate postprandial period. With respect to the physiological outcomes, the 400 mg dose elicited lower diastolic blood pressure and lower heart rate compared to the matched control, 1.5 h post-administration. These physiological benefits are likely related to previously observed vasodilatory [26] and glucoregulatory [28] effects of anthocyanins. The mechanisms for such physiological changes in response to flavonoid intervention are known to be dose dependent, and the current blood pressure and heart rate outcomes concur, with significant effects observed for the highest dose only. These blood pressure and heart rate changes are suggestive of potential physiological mechanisms of action for observed improvements to cognition, through increased blood flow to the brain and/or improved uptake of glucose to the brain. Previously, a reduction in systolic blood pressure was accompanied by improved episodic memory and visuospatial working memory following 3-month supplementation with a blueberry extract in healthy older adults [11]. Links have also been made between hypertension reduction and improvements to cognition [29, 30]. With respect to the current cognitive outcomes, the high dose (400 mg) elicited improvements to episodic memory, specifically AVLT word recall (R6) and recognition. Overall, however, the 200 mg dose elicited the majority of episodic memory effects, with improvements to immediate and delayed word recall at multiple recall points (R4, R5 and R7). The 100 mg dose elicited no AVLT benefits, suggesting that, as with the physiological effects, higher doses may be necessary to influence episodic memory.

Working memory effects were more equivocal, with only error rates influenced by the berry intervention, not overall scores. In the case of serial 3s, the 200 mg dose significantly reduced the number of errors compared to control; however, for serial 7s the 100 mg dose resulted in significantly fewer errors, while the 400 mg dose was observed to worsen performance. In the previous literature, differing effects for serial 3s and 7s have been observed following flavonoid-rich cocoa intervention in young adults [17]; the authors posit that the two levels of the task may test slightly different cognitive domains, with serial 7s drawing more heavily on executive function. However the difficulty of this subtraction task, particularly for serial 7s, may explain the mixed outcomes in this sample of older adults. Participants reported difficulty with both the working memory and data entry aspects of the task, which may have unduly confounded task performance irrespective of the berry dose. These working memory results should, therefore, be interpreted with caution. Participants reported no difficulty with the ANT executive function task; however, no significant effects were observed following any of the haskap berry doses. With respect to mood outcomes, there were no significant berry effects on positive affect, negative affect, or mental fatigue. However, alertness following the 400 mg dose was significantly lower than following the control. This observation remains unexplained but may be related to taste differences, particularly between the 400 mg dose and the control that remained evident despite efforts to match the doses for palatability. Specifically, the 400 mg dose was rated most sour, most bitter, least sweet, and least pleasant and, therefore, the poor taste of this dose may have impacted subjective perceptions of mood, or even cognitive performance. Olfactory stimuli have previously been observed to influence cognitive performance and mood [31]. However, this taste connection is purely speculative and requires further investigation. Taste differences may also have impacted the blinding of participants to the intervention; however, participants were only informed that the study was investigating the efficacy of a range of different haskap berry drinks, with no mention of a control or specific dose conditions. Therefore, the data are unlikely to have been unduly influenced by demand characteristics.

The findings broadly agree with previous literature. For the physiological outcomes, acute blood pressure changes have been observed following plum [32] and cherry supplementation [33]. Interestingly, plums, cherries and haskap berries all provide a rich source of cyanidin [13]. This specific anthocyanin compound forms a lower percentage of the anthocyanin profile in other berry fruits such as blueberries and blackcurrants, for which acute blood pressure changes are yet to be observed. Therefore, different fruits may provide different physiological benefits depending on their anthocyanin profile. Differential glucoregulatory effects of individual anthocyanin compounds have previously been reported [34]. Vasodilatory properties of individual compounds are yet to be investigated, but may also reveal compound-dependent differences in vascular response that would explain why acute blood pressure changes are evident following some berry fruits, but not others.

Review of the cognitive literature has revealed that acute flavonoid effects in older adults and children are more commonly related to memory rather than executive function [2]. Similar AVLT results to the current findings were observed in primary school children [8] and older adults [35] following acute blueberry supplementation. Acute working memory and executive function effects in older adults are not apparent in the berry flavonoid literature; however, acute working memory and attention benefits have been observed in this age group following curcumin supplementation [36]. In the current study, there were no executive function benefits observed on the ANT task, while the working memory effects proved difficult to interpret. Indeed, it is recommended that an alternative working memory task should be considered for inclusion in future older adult studies, as many participants found it difficult to navigate the computer related requirements of the task. The older adult sample was fairly heterogenous with respect to age, BMI, and habitual fruit and vegetable consumption, but is, therefore, representative of the wider ageing population. This was, however, a small sample size and so the cognitive benefits of haskap berries should be confirmed in a larger sample. The current findings are also limited to the immediate postprandial period and so it is recommended to investigate longer term supplementation to determine whether the memory benefits observed here translate to longer term benefits throughout the ageing process.

Finally, it should be noted that the study used a haskap berry extract as a concentrated source of anthocyanins, rather than whole berries. The doses here have been quantified as anthocyanins, but other polyphenols were also present in the extract (not quantified) so the outcome cannot solely be attributed to anthocyanins. This is often the case for whole food studies or those using extracts of whole foods. Here, the intervention was supplemented with fructose and glucose to mimic the sugar profile of the natural berries; however, it is also recommended to investigate the effects of whole haskap berries (in fresh or freeze-dried, powdered form), to confirm effect sizes in the presence of additional macronutrients such as fibre. The use of whole berries should also mitigate the taste issues created by the bitterness of the extract used in the current study.

In conclusion, haskap berry extract was observed to significantly improve postprandial episodic memory relative to a matched control, but showed limited effect on working memory or executive function. A significant lowering of diastolic blood pressure and heart rate relative to control was also observed, and is suggestive of a vasodilatory or glucoregulatory mechanism of action. Higher doses were observed to be more effective. Haskap berries may, therefore, be beneficial in ameliorating age-related memory deficits through improved vascular and metabolic health. As an extension to this work, studies investigating both the immediate postprandial and longer term cognitive benefits of whole haskap berries are recommended.
